# Exploring the Impact of Skin Care Routines on the Skin Microbiome and Possible Skin Disease Risk—A Pilot Study

**DOI:** 10.3390/biomedicines13102371

**Published:** 2025-09-27

**Authors:** Kirti Dubli, Preethi Balasundaram, Rinku Chaudhari, Sarvesh Vettrivelan, Arman Borawake, Raman Kapoor, Igor Kovalchuk, Anmol Kapoor, Raja Singh, Minal Borkar Tripathi

**Affiliations:** 1 Department of Biological Sciences, University of Lethbridge, 4401 University Drive, Lethbridge, AB T1K 3M4, Canada; 2 Department of Biological Sciences, University of Calgary, 2500 University Drive NW, Calgary, AB T2N 1N4, Canada; 3 BioAro Inc., Suite 1020 Calgary Tower Place, 1330—5th Avenue SW, Calgary, AB T2P 0C3, Canada

**Keywords:** skin, microbiome, sun exposure, sunscreen, moisturizer, atopic dermatitis, pigmentation, diet

## Abstract

**Background/Objectives:** Unceasing exposure of skin and its microbiome to various external and internal factors influences its health. Any imbalance ensuing may result in dysbiosis and consequently skin diseases. Thus, it becomes critical to identify and recognize the influence of several factors on the skin microbiome and various disorders associated with it. **Methods:** In the current investigation, we studied the skin microbiomes of 37 subjects using the next-generation shotgun sequencing method and compared them with 48 healthy subjects from the Human Microbiome Project (HMP). This work focused on the analysis of the impact of different skin care routines—use of sunscreen and moisturizers—on the skin microbiome and related skin diseases. **Results:** Differences were observed between the microbiomes of subjects who were using only moisturizer (*p* = 3.1 × 10^−5^) or moisturizer with sunscreen (*p* = 3.2 × 10^−13^) and those who did not use these products at all. We also observed differences in the skin microbiomes of males vs. females with respect to the use of moisturizer. The composition of the skin microbiomes of female participants showed a higher difference in diversity in comparison to males. The current investigation also found that usage of sunscreen might help in retaining skin-protecting species in the skin microbiome. **Conclusions:** This work allowed us to understand the impact of moisturizer and sunscreen on skin health. The present evaluation shall pave the way for personalized skin care product development.

## 1. Introduction

Healthy skin illustrates the well-being of the human body. Skin is the largest organ of the human body, shielding its interior from various external factors like sun exposure—specifically UV radiation—chemicals, infection, and external injury [1,2]. It also functions as the primary shield of the immune system. The skin comprises different layers—the epidermis, dermis, and hypodermis. Microorganisms are also associated with these different skin layers. Skin microorganisms and their gene expressions work in conjunction with genes expressed by host skin cells to sustain an intricate association with the skin, including the skin microbiome [3]. The skin microbiome is critical to the human skin surface, as it participates in inhibiting pathogen growth, developing innate immunity and adaptive immunity by reducing inflammation post-injury, regulating the production of local cytokines, etc. [4,5].

Multifunctional skin microbiome is influenced by age, lifestyle, etc. Work by Nagase et al. showed that bedridden older individuals have a distinct microbiome and are more prone to infections when compared to young subjects [2]. Additionally, the skin microbiome is influenced by cosmetics. A study by Hwang et al. demonstrated that the application of cosmetics on the skin led to an increase in the relative abundance of *Cutibacterium* spp. and *Staphylococcus* spp. in the skin microbiome [6]. Furthermore, a previous study by Wallen-Russell et al. also showed that synthetic ingredients from cosmetics influence the diversity of the skin microbiome [7]. Likewise, cosmetics rich in *Ginkgo biloba* extract, lavender oil, and components from other natural ingredients like almond reduce wrinkles [8]. Work by Hekmatpou et al. showed *Aloe vera* and its compounds facilitated the retention of skin moisture and supported skin integrity [9].

Humans use cosmetics like moisturizer, sunscreen, etc., daily to enhance their appearance and improve their well-being. Moisturizers refer to those substances that, when applied to the skin, add water to the skin surface and increase the water-binding capacity of the stratum corneum of the skin and maintain its integrity [10]. As moisturizers allow skin moistening, they benefit skin by their anti-inflammatory, anti-pruritic, wound-healing, and anti-mitotic properties [11]. For instance, moisturizers are extensively utilized for conditions like atopic dermatitis [12]. Thus, it becomes important to understand if the usage of moisturizer on a routine basis can help in mitigating the possible disease risk for conditions like atopic dermatitis. Furthermore, the skin is prone to damage by UV-A and UV-B radiation, which may result in premature aging or cancer due to DNA damage and oxidative stress. A study on sun exposure conducted on inhabitants of the Mediterranean coast demonstrated the absence of the taxa *Streptococcaceae* and *Cyanobacteria*, specifically in summers [13]. Another cosmetic used for skin protection from sun exposure is sunscreen. Sunscreens refer to those cosmetics that protect skin from UV damage and photodamage [14,15]. Usage of sunscreens prevents sunburn, photoaging, freckles, hyperpigmentation, etc. [16,17].

Diet and intestinal/gut microbiome also influence skin health [18]. For example, a diet rich in packaged and processed foods has been associated with the development of skin conditions like atopic dermatitis and psoriasis [19]. Additionally, the consumption of mangoes and almonds is linked with the reduction of wrinkles, while the consumption of avocados is associated with increased firmness and elasticity of the skin. Furthermore, a plant-based diet has shown positive effects on inflammatory diseases like psoriasis, acne, and atopic dermatitis [20]. Moreover, an investigation by Ying et al. on the correlation between age and gender in urban and rural populations with respect to skin microbiome in Shanghai, China, demonstrated the higher abundance of *Trabulsiella* in the urban population. Furthermore, the adults showed higher microbial diversity when compared to teenagers or older individuals [21]. Extensive studies have been conducted on understanding the effect of various factors on skin and skin microbiome, but very little work has been performed in integrating the skin care routine, sun exposure, and type of diet with the skin microbiome and possible risk of related skin disorders—atopic dermatitis and hyperpigmentation. Hence, the current study explores the potential of the use of moisturizer and sunscreen and its association with the development of conditions like atopic dermatitis and hyperpigmentation with skin microbiome. Likewise, this investigation aims to explore the impact of the type of diet consumed on skin microbiome variation.

## 2. Materials and Methods

### 2.1. Study Population and Design

This study was approved by the Health Research Ethics Board of Alberta (HREBA)—Community Health Committee (CHC) with ethics ID: HREBA.CHC-25-0013 (approval date: 15 May 2025). This cross-sectional retrospective study comprised 37 subjects (27 females and 10 males). All the participants included in this study provided informed consent prior to providing their sample. Skin microbiome testing was performed at BioAro Inc. Hence, BioAro Inc. has a database of skin microbiome results. The skin microbiome samples from facial skin were collected by using the BioSkin kit from BioAro Inc. (Calgary, AB, Canada) as per the manufacturer’s instructions. Briefly, the swab was dipped in the wetting solution and rubbed on the surface of the cheek for 30 s. The swab was then transferred to the collection tube provided. The samples were stored at −20 °C until further use.

Information about the sun exposure (number of hours exposed to the sun daily), use of moisturizer (routine and frequency of use daily), sunscreen (routine and frequency of use daily), and type of diet (plant-based or meat-based) was also compiled from the BioAro Data repository. This study excluded the subjects who were pregnant or lactating and subjects who had undergone skin treatment or antibiotic treatment within 3 months prior to the sequencing. The subjects were instructed to wash their face overnight before the sample collection and not to apply any cosmetics 6 h preceding sample collection, as the chemicals in the cosmetics interfere with the skin microbiome sample collection and affect the DNA extraction process. Only those subjects for whom all the information regarding moisturizer, sunscreen use, sun exposure, and type of diet was available were selected for this study. Data for 48 healthy subjects were collected from the HMP site (website URL: https://commonfund.nih.gov/hmp; accessed on 25 May 2025). The participants’ characteristics are shown in Table 1.

### 2.2. Extraction of DNA

The microbiome DNA was extracted using the ZymoBiomics DNA Miniprep kit (Zymo Research, Corp., Irvine, CA, USA). Briefly, the sample was lysed using the lysis solution from the manufacturer, followed by bead beating and DNA purification by spin columns as per the manufacturer’s protocol. Briefly, 250 μL of sample and 750 μL of lysis solution were added to ZR BashingBead™ Lysis Tubes (Zymo Research, Corp., Irvine, CA, USA) provided in the kit, followed by bead beating. Centrifuge the tubes at ≥10,000× *g* for 1 min. Furthermore, 400 μL of supernatant was transferred to a Zymo-Spin™ III-F Filter in a Collection Tube and centrifuged at 8000× *g* for 1 min. The filtrate is now mixed with 1200 μL of ZymoBIOMICS™ DNA Binding Buffer. The mixture was transferred to spin columns and centrifuged at 10,000× *g* for 1 min, followed by a subsequent wash with wash buffer. The DNA was eluted using 100 μL of ZymoBIOMICS™ DNase/RNase Free Water provided in the kit. The extracted DNA was quantified on a Qubit Fluorometer 3.0 (Thermo Fisher Scientific, Waltham, MA, USA).

### 2.3. Next-Generation Sequencing

The extracted DNA samples were processed for paired-end shotgun sequencing by the next-generation sequencing method. A library of the samples (350–450 nucleotides) was generated on MGI-SP100 (MGI Tech Co., Ltd., Shenzhen, China) according to the manufacturer’s instructions. Briefly, 200 ng of the extracted DNA was used as input for the process. The extracted DNA was ligated to the adaptors with barcodes from the MGIEasy FS DNA Library Prep Set (MGI Tech Co., Ltd., Shenzhen, China), followed by amplification to generate libraries. The library was quantified using a Qubit 3.0 Fluorometer using a Qubit 1X HS dsDNA assay kit, and the fragment size was checked using an Agilent TapeStation. The library of all samples was then circularized using the MGIEasy Circularization Module (MGI Tech Co., Ltd., Shenzhen, China). The adapter-ligated library was circularized to an ssDNA library. This circularized library was then loaded into the flow cells for post-processing using the DNBSeq-G400RS High Throughput Sequencing Kit (MGI Tech Co., Ltd., Shenzhen, China) and DNBSeq-G400RS Sequencing Flow Cell (MGI Tech Co., Ltd., Shenzhen, China) on the MGI-DNBG400 sequencer (MGI Tech Co., Ltd., Shenzhen, China). Negative control was included throughout the procedure for detecting material and reagent contaminations.

### 2.4. Analysis of the Microbiome

For the analysis of skin microbiome data, we used a standardized in-house bioinformatics pipeline that chiefly applied Bash and the R programming language. Raw sequencing reads in FASTQ format were provided as input into a two-step preprocessing workflow, which was intended to ensure high-quality, host-depleted microbial data. Initially, Cutadapt (version 4.1) [22] was utilized to remove adapter sequences and trim bases of low quality. Trimming parameters were adjusted to retain reads with a minimum length of 150 base pairs and a Phred quality score of at least Q30 to reduce the influence of sequencing artifacts in downstream analyses of sequencing data. Quality metrics were assessed with the help of FastQC (Version 0.12.0) [23], which provided per-base quality profiles, duplication rates, GC content distribution, and other sequencing quality indicators.

Subsequently, reads obtained from the host organism were filtered using KneadData (version 0.12.3) [24], which aligns reads against the human reference genome (GRCh38) using Bowtie2 [25]. Later, reads that were mapped to the host genome were removed to minimize host contamination of human DNA, as it can constitute a substantial portion of total reads. Microbial taxonomic profiling was performed using MetaPhlAn 4 [26,27], which depends on a curated set of clade-specific marker genes for accurate taxonomic classification from metagenomic reads. Downstream ecological and statistical analyses, including alpha and beta diversity assessments, differential abundance testing, and data visualization, were performed in R (version 4.3.1) with custom RMarkdown scripts to ensure complete reproducibility.

### 2.5. Statistical Analysis

R studio (version 2024.04.0-735) was used to perform tertiary analysis. A heatmap of the relative abundance of different species was prepared using the heatmap.2 of the ggplot2 package. The Kruskal–Wallis test, Wilcoxon test, *t*-test, and ANOVA were used to compare the participants’ characteristics and relative abundance amongst the groups. Intergroup and subgroup analysis was performed to compare variations within the groups.

## 3. Results

### 3.1. Microbiome Composition and Alpha Diversity

**Figure 1:**
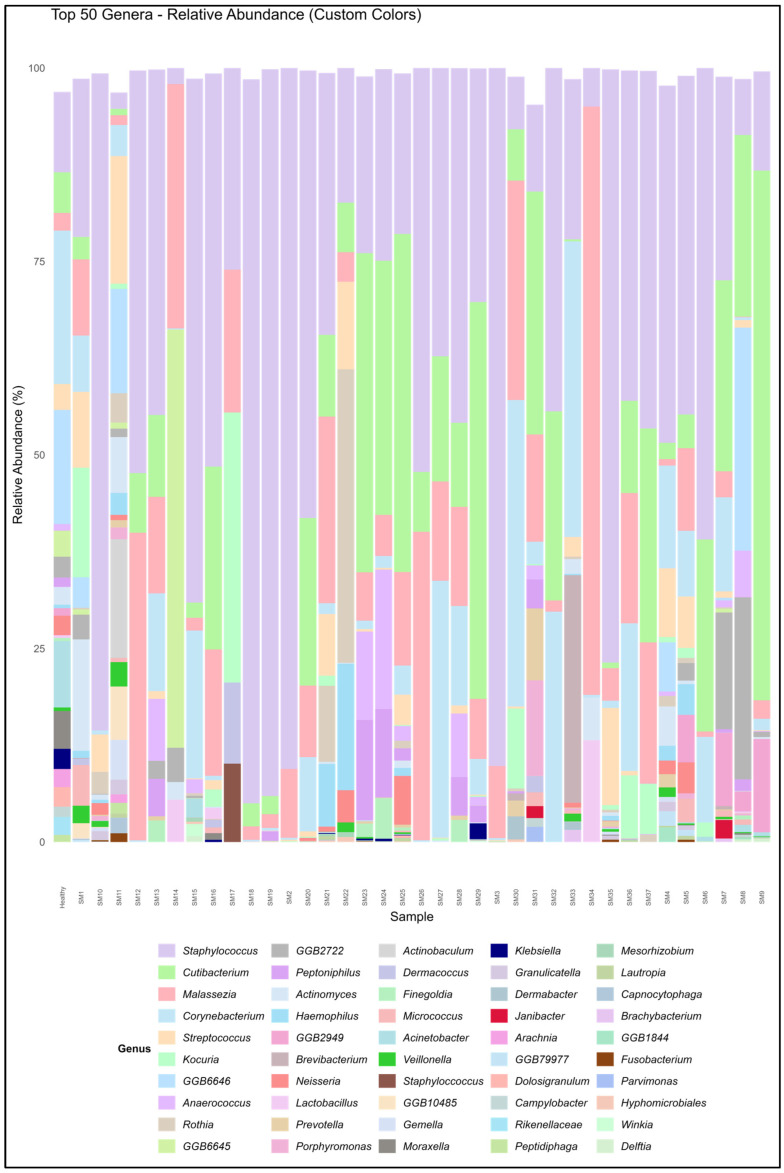
Relative abundance of top 50 genera for all participants at the genus level across all samples.

**Figure 2:**
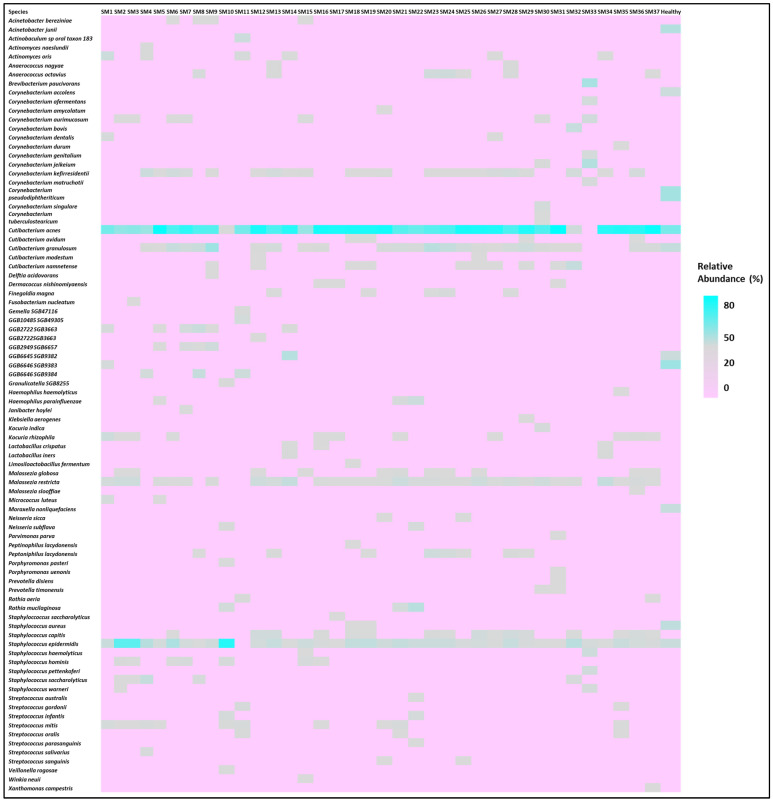
Heat map of majorly found species for all the subjects, showing variation in the species observed.

The participant characteristics are depicted in Table 1. Table 2 shows their Shannon diversity and genus-level relative abundance of *Cutibacterium* spp., *Streptococcus* spp., *Corynebacterium* spp., *Staphylococcus* spp., and *Malassezia* spp. Moreover, 10 males and 27 females were part of this study. In this study, 32.43% of the participants had oily skin, while the rest of the participants had dry skin. All the participants used moisturizers except for five. Participants were also classified based on daily sun exposure. Moreover, the Shannon Diversity was calculated for skin microbiome samples for all the participants. The diversity values varied from 0.21 to 2.73 for the participants in this study. The microbiome composition of a few major species is depicted in Figure 1, which shows the relative abundance of the top 50 species for the participants. This assessment was made after all the sequences were rarefied at 15,580 depths. The heat map of the top 10 species for all participants is shown in Figure 2. All the subjects in this study were compared with the 48 healthy subjects from the HMP site (data included in Table 2). *Cutibacterium acnes* and *Staphylococcus epidermidis* were observed in all the participants.

### 3.2. Relationship Between Skin Care Routine and Skin Microbiome

**Figure 3:**
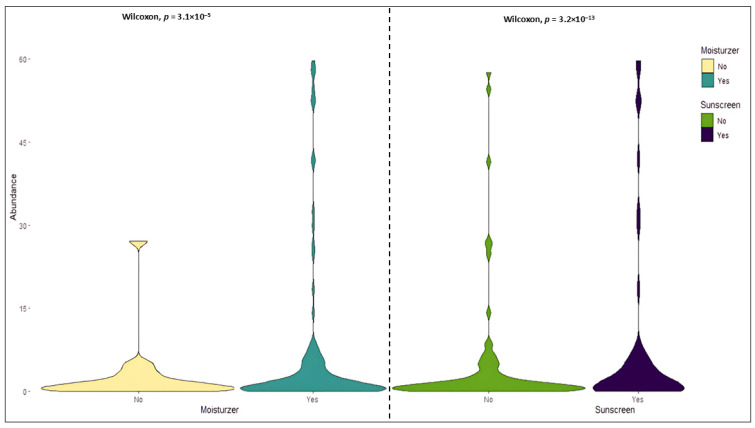
Relative abundance of microbiome based on the use of moisturizers among the subjects; the difference in the relative abundance of subgroups based on the usage of moisturizers (*p* = 3.1 × 10^−5^) and sunscreens (*p* = 3.2 × 10^−13^) is significant (subjects using moisturizer = 30, subjects not using moisturizer = 7; subjects using sunscreen = 23, subjects not using sunscreen = 14).

All the participants in this study were subgrouped according to usage of moisturizer, sunscreen, daily sun exposure, and type of diet. The comparison between the subjects using moisturizer and sunscreen was significant (Wilcoxon test *p* = 3.1 × 10^−5^, *p* = 3.2 × 10^−13^) as depicted in Figure 3.

**Figure 4:**
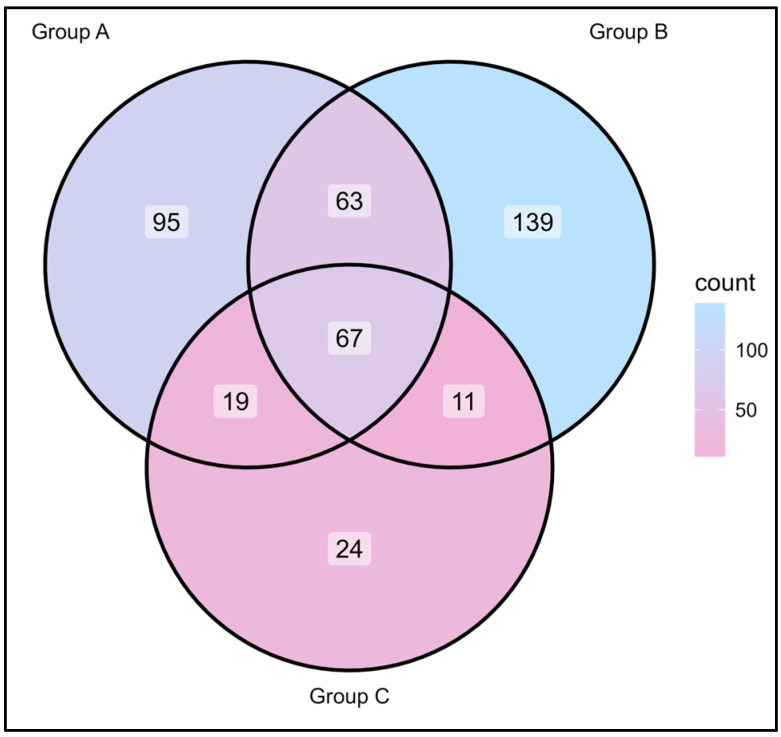
Venn diagram at the microbial level from participants using moisturizer and sunscreen (Group A, *n* = 23), participants using only moisturizer (Group B, *n* = 7), and participants not using moisturizer and sunscreen (Group C, *n* = 7).

*Cutibacterium acnes* and *Staphylococcus epidermidis* were the major species found in all samples, irrespective of the skin care routine or gender of the participants. Figure 4 depicts the common and uncommon microorganisms observed in the skin microbiota of the different groups of participants based on their skin care routine (use of moisturizer and sunscreen).

**Figure 5:**
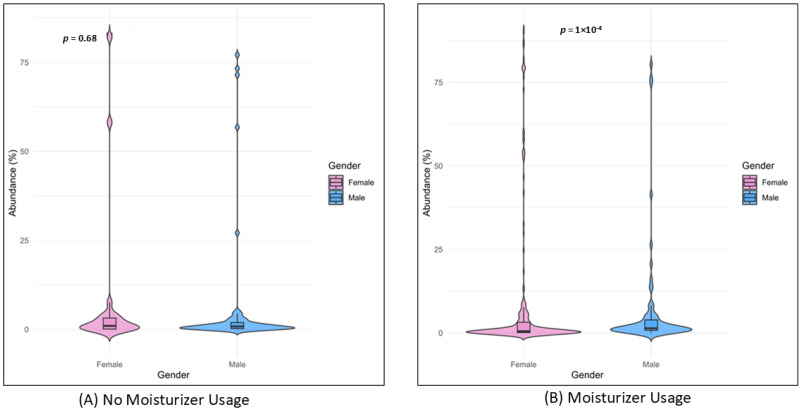
Relative abundances of the top 10 species in females and males. (**A**) No moisturizer usage in females (*n* = 2) and males (*n* = 3) was non-significant (*p* = 0.68). (**B**) Moisturizer usage in females (*n* = 25) and males (*n* = 8) was highly significant (*p* = 1 × 10^−4^).

*Corynebacterium sanguinis* and *Brachybacterium nesterenkovii* were observed in participants using both moisturizer and sunscreen, while *Corynebacterium kroppenstedtii* and *Micrococcus terreus* were seen in participants with no skin care routine. *Corynebacterium kefirresidentii*, *Cutibacterium avidum,* and *Streptococcus capitis* were also found to be common among all three groups. Furthermore, male and female participants were compared based on their skin care routine, i.e., based on the use of moisturizer. The relative abundance and diversity were significantly higher in females with respect to the males who used moisturizer (*p* = 1 × 10^−14^), while it was non-significant for males and females not using moisturizer, as observed in Figure 5.

Higher relative abundances and diversity are observed in females. The high significance observed could be attributed to two reasons: (1) the number of male participants is less than female participants; (2) there are differences in the skin microbiome of male and female participants, irrespective of skin care routine. This trend can be more clearly understood if we compare the male and female participants who follow a skin care routine and those participants who do not follow a skin care routine, using a larger cohort size.

### 3.3. Skin Microbiome and Sun Exposure

**Figure 6:**
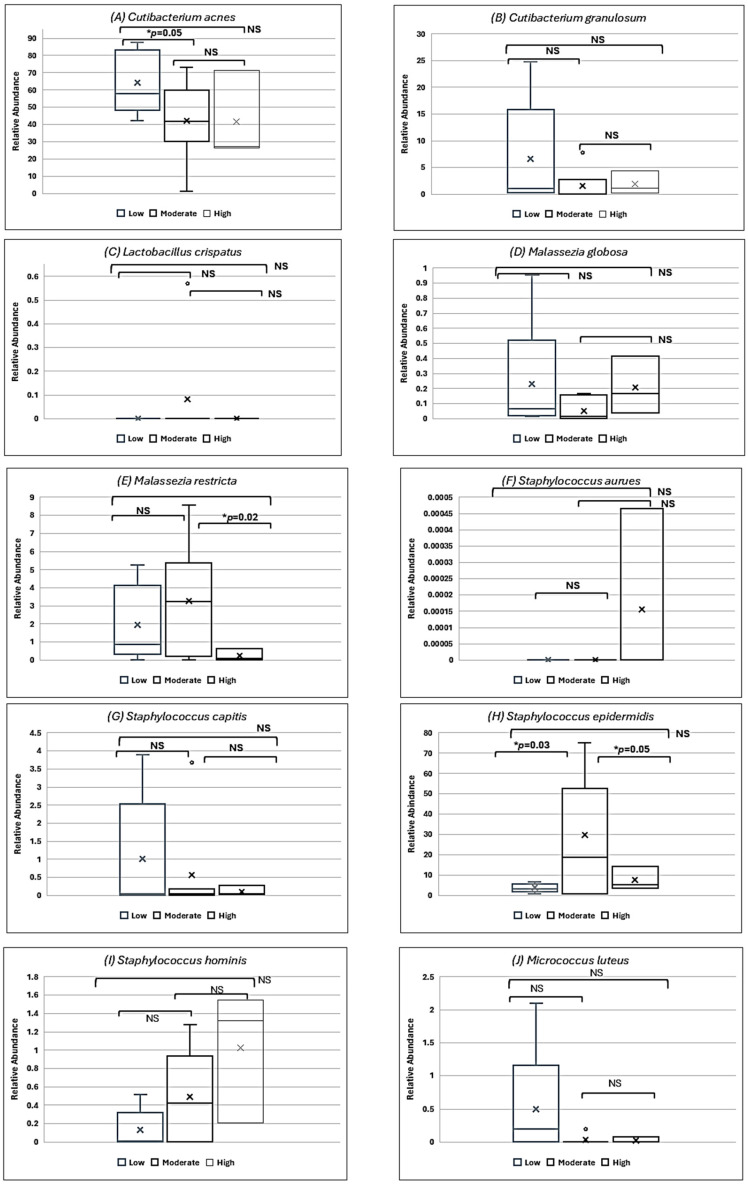
Relative abundance of some commonly seen species in all samples compared with daily exposure to sun: (**A**) *Cutibacterium acnes*, (**B**) *Cutibacterium granulosum*, (**C**) *Lactobacillus crispatus*, (**D**) *Malassezia globosa*, (**E**) *Malassezia restricta*, (**F**) *Staphylococcus aureus*, (**G**) *Staphylococcus capitis*, (**H**) *Staphylococcus epidermidis*, (**I**) *Staphylococcus hominis,* and (**J**) *Micrococcus luteus.* Comparisons were found to be significant for *C. acnes* (low vs. moderate sun exposure), *M. restricta* (moderate vs. high sun exposure), and *S. epidermidis* (low vs. moderate sun exposure; moderate vs. high sun exposure). The box plot center line indicates the median; ‘x’ represents the mean; and ‘o’ represents the outliers. The edges of boxes are quartiles, and the error bar depicts the max. value. Note: NS = not significant; * *p* = significant value.

The participants were also grouped based on the sun exposure shown in Table 1. The relative abundance of the species was compared for a few bacteria prominently observed in all participants grouped by their skin care routine. The box plots for them are shown in Figure 6. Significant associations were found in *C. acnes* (low vs. moderate sun exposure, *p* = 0.05), *Malassezia restricta* (moderate vs. high sun exposure, *p* = 0.02), and *S. epidermidis* (low vs. moderate sun exposure, *p* = 0.03; moderate vs. high sun exposure, *p* = 0.05).

### 3.4. Skin Microbiome, Moisturizer and Atopic Dermatitis

**Figure 7:**
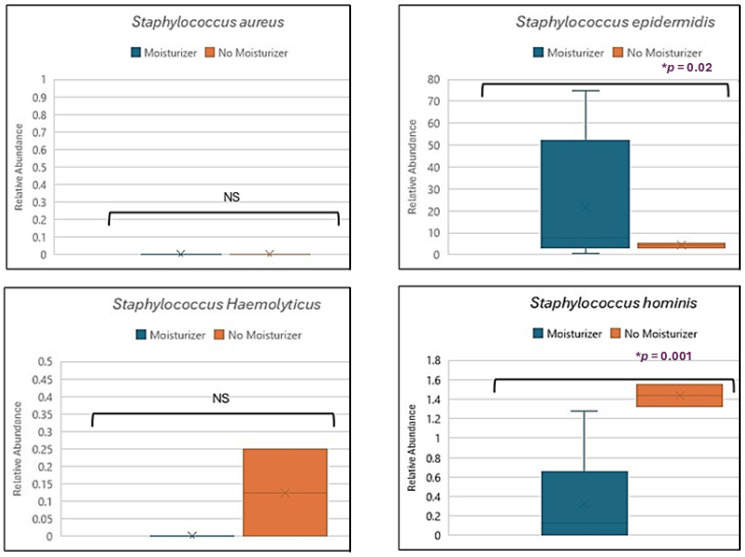
Relative abundance comparison for *Staphylococcus aureus*, *Staphylococcus epidermidis*, *Staphylococcus haemolyticus,* and *Staphylococcus hominis* in subjects using moisturizer and no moisturizer. It was found to be significant in *S. epidermidis* and *S. hominis* based on the usage of moisturizer. The box plot center line indicates the median; ‘x’ represents the mean; the edges of boxes are quartiles; and the error bar depicts the max. value. Note: NS = not significant; * *p* = significant value.

The current study explored the impact of sunscreen on *Staphylococcus aureus*, *S. epidermidis* (*p* = 0.02), *S. haemolyticus,* and *S. hominis* (*p* = 0.001) species of bacteria, which are associated with atopic dermatitis, as depicted in Figure 7.

### 3.5. Skin Microbiome, Sunscreen, and Hyperpigmentation

**Figure 8:**
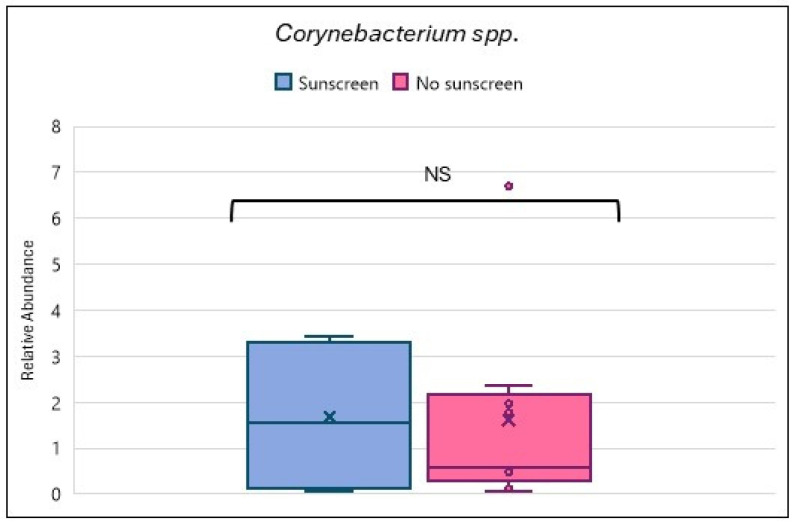
Relative abundance comparison for *Corynebacterium* spp. for subjects using sunscreen and no sunscreen. The box plot center line indicates the median; ‘x’ represents the mean; ‘o’ represents the outliers; the edges of boxes are quartiles; and the error bar depicts the max. value. Note: NS = not significant.

This study also attempted to analyze the effect of sunscreen use on *Corynebacterium* species associated with hyperpigmentation, as shown in Figure 8. No significant difference was noticed.

### 3.6. Skin Microbiome and Plant-Based Diet

**Figure 9:**
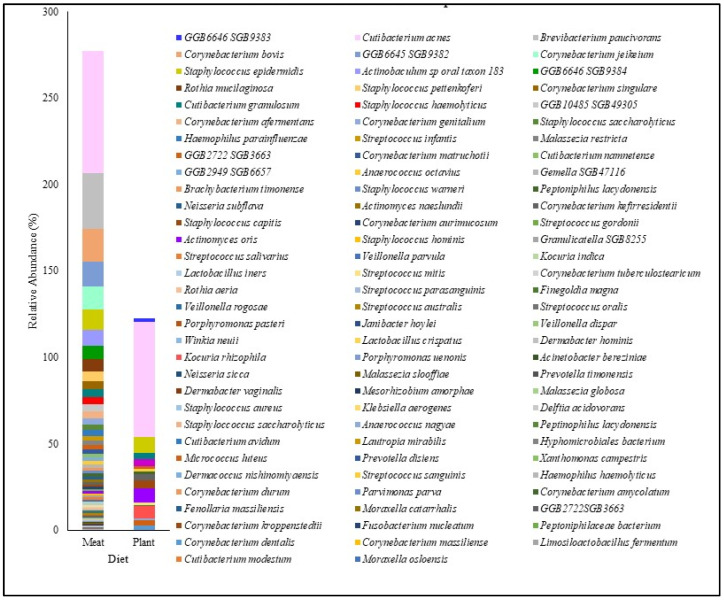
Relative abundance comparison of microbiome based on a meat-based diet and a plant-based diet for participants. The comparison was highly significant with *p* = 0.0001.

This study also investigated the difference in the skin microbiome of subjects using moisturizer and sunscreen according to a plant-based diet and a meat-based diet consumed, as shown in Figure 9. However, we do not have an equal number of participants who consumed a plant-based diet (*n* = 3) and the meat-based diet (*n* = 34). The analysis yielded higher diversity in subjects with a meat-based diet, while it was lower in subjects with a plant-based diet (*p* = 0.0001).

## 4. Discussion

Skin is constantly exposed to various physical, chemical, and biological factors that affect the skin and its microbiome [28]. The skin microbiome plays an active role in skin health. The microbiota on the surface of skin stimulates the host skin cells to release antimicrobial peptides, inhibits pathogens, and, moreover, helps in the differentiation of T-cells. Any change in the healthy balance of the skin microbiome may lead to dysbiosis, resulting in various inflammatory disorders of the skin. Thus, it is important to understand and fathom the effects of various internal and external factors on the skin microbiome [29]. In the current investigation, we focused on integrating the effect of skin care routine and type of diet in conjunction with the sun exposure and risk of skin disorders (atopic dermatitis and hyperpigmentation) with the skin microbiome. Earlier studies showed that skin health is affected by numerous factors leading to the development of the skin interactome [30]. The skin interactome is based on integrating the knowledge regarding the microbiome, genome, and exposome. Current analysis shall result in improved understanding of the skin interactome as it focuses on assimilating and incorporating the effect of multiple factors like skin care routine, diet, and sun exposure influencing skin health and microbiome.

This analysis allowed us to identify the differences in the skin microbiome based on skin care routine—use of moisturizer and sunscreen, sun exposure, and type of diet consumed—plant-based or meat-based. Investigating the effect of skin care routines in participants indicated that the relative abundance of species in the skin microbiome was higher in participants using only moisturizer compared to participants using both moisturizer and sunscreen. Comparison of various groups based on skin care routine recognized the common species among all groups, like *C. acnes*, *S. epidermidis*, *C. kefirresidentii*, *C. avidum,* and *S. capitis.* An earlier 16S rRNA-based study of the diversity of the microbiome of skin identified similar genera common to the skin, which were *Cutibacterium*, *Streptococcus,* and *Staphylococcus* spp. [6,30].

Earlier studies reported the effect of cosmetics on skin microbiome as a function of age and skin condition [21]. Our study is one of the initial studies that differentiates between the skin microbiome of males and females according to skin care routine. The current examination detected higher abundance of bacteria like *S. epidermidis*, *M. restricta*, and *C. acnes* in female participants. A previous study conducted on skin microbiomes from axillary regions in males and females showed that *Corynebacterium amycolatum* and *Corynebacterium kroppenstedtii* were specifically identified in males, while *Corynebacterium urealyticum* and *Corynebacterium variabile* were only seen in females [31]. This was slightly contradictory to the results of the current study, as *C. amycolatum* was found in the facial skin microbiome of both male and female participants. Moreover, *C. urealyticum* and *C. variabile* were not found in female participants.

Sun exposure is critical to the health of the skin. Exposure to ultraviolet radiation can lead to various skin problems like sunburn, skin inflammation, early aging, or skin cancer. As per the recommendation by the American Academy of Dermatology, using sunscreen, avoiding high exposure to the sun, using protective clothing, and avoiding tanning are considered good practices for the prevention of skin cancer [32,33,34]. In an independent time-point study conducted on holidaymakers, it was observed that the sun exposure reduced the proteobacteria in study participants. These proteobacteria were restored to their pre-holiday levels after 28 days [34]. Studies conducted on the effects of UV radiation and *Malassezia* spp. showed that these microorganisms produce pityriacitrin, which helps in the protection of host and microbial cells [35]. Investigation of UV radiation on murine skin microbiome discovered that *S. epidermidis* protected the host from chronic exposure [36]. Examination of the effect of UV exposure on skin microbiome in the presence of sunscreen found that *C. acnes* was abundant in all subjects with exposed, non-exposed, placebo, and sunscreen-covered skin [37]. In the present analysis, we compared the relative abundance of *C. acnes*, *C. granulosum*, *L. crispatus*, *M. globosa*, *M. restricta*, *S. aureus*, *S. capitis*, *S. hominis*, *S. epidermidis,* and *M. luteus*. These species were considered due to their association with skin and sun exposure. Our analysis found that sun exposure was found to have a significant effect on the abundance of *C. acnes* (low vs. moderate sun exposure), *M. restricta* (moderate vs. high sun exposure), and *S. epidermidis* (low vs. moderate sun exposure and moderate vs. high sun exposure). Prior studies conducted showed that *C. acnes* abundance is affected in the presence of sunscreens [38], which corroborates with observations made in this study. Furthermore, the presence of *M. restricta* and *S. epidermidis* observed in all participants in this study, irrespective of sun exposure, is indicative of their essential role in protecting the host skin.

Hyperpigmentation of the skin can arise due to multiple factors, one of which includes sun exposure [37]. This type of hyperpigmentation includes melasma and post-inflammatory hyperpigmentation. Hyperpigmentation can result in early photoaging and senescence of the skin melanocytes [39]. A prior study conducted by Fatima et al. showed that usage of sunscreen functioned as an adjuvant in therapy for hyperpigmentation and aided in improvement [17]. Hyperpigmentation on skin is linked to *Corynebacterium* spp. [40]. In this current investigation, no significant correlation was observed between *Corynebacterium* spp. and use of sunscreen. This may be attributed to the small cohort size. A larger cohort size might provide better insight into the association between hyperpigmentation, *Corynebacterium* spp., and sunscreen use.

Atopic dermatitis is a commonly observed skin disorder. It is an inflammatory disorder affecting all age groups [41]. Skin microbiome is fundamental from the perspective of atopic dermatitis, as it is severely affected by this disorder. Infection of *S. aureus* can exacerbate skin damage and result in dysbiosis [42]. This disorder is characterized by dry and itchy skin, recurring eczema flares, and immune dysregulation stimulating production of the antibody IgE [43]. Atopic dermatitis is linked with colonization of *S. aureus* [17,41,42,43,44] and increased abundance of *S. haemolyticus* [38]. However, *S. epidermidis* and *S. hominis* are associated with providing protection against pathogens and preventing dysbiosis of the skin microbiome [42,44,45]. These bacterial species produce antimicrobial compounds against *S. aureus*, thus preventing its colonization [45]. In addition, the treatment steps in atopic dermatitis involve using moisturizer to nourish the dry skin patches [46]. Our study attempted to check the effect of the use of moisturizer in enhancing skin protective species like *S. epidermidis* and *S. hominis*. Our results supported the hypothesis that using moisturizer enhanced the growth of skin-protecting species like *S. epidermidis* and *S. hominis,* which prevent or interfere with the growth of pathogens like *S. aureus*.

Food is a source of energy and nutrients for the human body, and it is critical for overall health, including the skin [47]. Skin health is influenced by both external and internal factors. One of the major internal factors influencing skin is the type of diet consumed. Recently, there was effort in understanding the effect of type of diet on skin health [48]. The type of diet consumed is crucial to the gut microbiome. Dysbiosis of the gut microbiome is linked to various skin disorders like atopic dermatitis, rosacea, acne, and psoriasis. Numerous studies conducted previously have linked the health of skin to homeostasis of the gut [49]. Prior studies demonstrated that the phenol and p-cresol produced by *Clostridiodes difficile* in the gut are associated with reduced moisture in the skin, consequently affecting keratinization and skin barrier integrity [50]. Thus, the gut–skin axis becomes one of the major criteria to understand lifestyle impact on skin. Previous research indicated that atopic dermatitis [51], psoriasis, and rosacea [52] might be linked to the gut–skin axis. In the current investigation, we compared the type of diet in participants. Our analysis had limitations of a small sample size, and using a larger cohort size will help in better comprehension of the trends or effect. The small size of the plant-based diet in comparison with the meat-based diet was primarily due to the lower number of participants used in this study from the BioAro data repository after selecting the subjects based on inclusion–exclusion criteria. However, we detected the presence of unique species in plant-based diets, such as *Veillonella parvula*, *Streptococcus mitis*, *Streptococcus cristatus*, *Mesorhizobium amorphae*, *Micrococcus luteus*, *Kocuria rhizophila*, *Gemella hemolysans*, and *Actinomyces oris*. This is primary research on the difference in skin microbiota of participants following a skin care routine but consuming distinct types of diets. Furthermore, our study comprised a relatively smaller group of males when compared to females, as it was obtained retrospectively from the BioAro database repository, which could give rise to potential non-comparability and must be ascertained with the help of a larger dataset. A detailed investigation with a large cohort size will be beneficial in developing the knowledge of the gut–skin axis from the perspective of skin care practices and analysis of the risk of various skin disorders by improving dietary habits.

## 5. Conclusions

Skin and its microbiome are constantly exposed to various physical, biological, and chemical factors that may affect skin health positively or negatively [53]. Prior to the 16S rRNA-based sequencing study on skin metabolomes of a small group of subjects, it was confirmed that the skin care routine influences the chemistry of the skin and its microbiome [54]. The results of the current study are primarily centered on evaluating the effect of skin care routine, sun exposure, and type of diet consumed on skin microbiome at the species level of diversity. This work efficaciously proved the potential of these factors in influencing the skin microbiome. Skin care routines and sun exposure concomitantly impact the skin microbiome. Though the exact molecular mechanism of the ensuing effect is not known, this study shall provide future guidance in advancing the medicine for healthy glowing skin.

## Figures and Tables

**Table 1:** Participants’ characteristics.

Sample ^$^	Gender	Skin Type	Diet	Moisturizer Use	Sunscreen Use	Sun Exposure *
SM1	Female	Oily	Plant	Yes	Yes	Low
SM2	Female	Dry	Meat	Yes	Yes	Moderate
SM3	Female	Dry	Meat	Yes	Yes	Moderate
SM4	Male	Dry	Meat	Yes	No	High
SM5	Female	Dry	Meat	Yes	No	Low
SM6	Female	Dry	Meat	Yes	Yes	Moderate
SM7	Male	Dry	Meat	No	No	High
SM8	Female	Oily	Meat	Yes	No	Low
SM9	Female	Dry	Meat	Yes	No	Low
SM10	Male	Dry	Meat	Yes	No	Moderate
SM11	Male	Oily	Meat	Yes	No	Moderate
SM12	Female	Oily	Meat	Yes	Yes	Low
SM13	Female	Oily	Plant	Yes	Yes	Moderate
SM14	Female	Dry	Meat	Yes	No	Moderate
SM15	Male	Dry	Meat	No	No	High
SM16	Female	Oily	Meat	Yes	Yes	Low
SM17	Female	Oily	Meat	Yes	Yes	Low
SM18	Female	Dry	Meat	Yes	Yes	Moderate
SM19	Female	Dry	Meat	Yes	Yes	Moderate
SM20	Female	Dry	Meat	No	No	Moderate
SM21	Female	Dry	Meat	Yes	Yes	Moderate
SM22	Female	Dry	Meat	Yes	Yes	Low
SM23	Female	Oily	Meat	Yes	Yes	Moderate
SM24	Female	Oily	Meat	Yes	Yes	Moderate
SM25	Male	Dry	Meat	Yes	Yes	Low
SM26	Female	Oily	Meat	Yes	Yes	Moderate
SM27	Male	Dry	Meat	No	No	High
SM28	Female	Oily	Plant	No	No	Moderate
SM29	Male	Dry	Meat	Yes	Yes	Low
SM30	Male	Dry	Meat	No	No	High
SM31	Female	Dry	Meat	Yes	Yes	Low
SM32	Male	Dry	Meat	Yes	Yes	Moderate
SM33	Male	Dry	Meat	Yes	Yes	Moderate
SM34	Female	Dry	Meat	Yes	Yes	Low
SM35	Female	Oily	Meat	Yes	Yes	Moderate
SM36	Male	Dry	Meat	No	No	High
SM37	Female	Oily	Meat	Yes	Yes	Moderate

^$^ Sample – SM; * Sun exposure—Low = less than 1 h; Moderate = 1–3 h; High = more than 3 h daily.

**Table 2:** Shannon diversity and total relative abundance of a few key genera.

Sample	Shannon Diversity	*Corynebacterium* spp.	*Cutibacterium* spp.	*Staphylococcus* spp.	*Malassezia* spp.
SM1	2.12	2.90 ± 0.73	43.13 ± 19.52	8.15 ± 2.68	3.92 ± 1.01
SM2	0.93	0.15 ± 0.00	29.86 ± 0.00	53.50 ± 22.57	5.25 ± 2.47
SM3	0.94	0.16 ± 0.01	32.48 ± 0.01	53.25 ± 24.78	5.51 ± 2.59
SM4	2.4	6.62 ± 2.34	27.46 ± 12.68	23.03 ± 5.96	0.41 ± 0.17
SM5	0.43	0.57 ± 0.12	87.61 ± 41.10	2.93 ± 1.36	0.72 ± 0.27
SM6	1.02	3.43 ± 1.63	67.36 ± 25.99	18.88 ± 8.58	0.21 ± 0.09
SM7	1.04	2.35 ± 0.48	76.24 ± 26.32	5.09 ± 1.30	0.65 ± 0.29
SM8	1.32	0.10 ± 0.05	64.29 ± 25.71	2.07 ± 0.48	0.02 ± 0.01
SM9	1.14	0.54 ± 0.14	79.54 ± 21.52	4.69 ± 0.01	0.87 ± 0.41
SM10	0.99	0.47 ± 0.05	1.00 ± 0.01	76.10 ± 34.95	0.00 ± 0.00
SM11	2.73	1.49 ± 0.26	41.76 ± 19.43	0.78 ± 0.34	0.47 ± 0.23
SM12	0.61	0.03 ± 0.00	80.05 ± 34.07	6.97 ± 0.40	5.32 ± 2.59
SM13	1.27	3.24 ± 1.59	60.88 ± 26.80	11.47 ± 3.04	3.20 ± 0.00
SM14	0.91	0.04 ± 0.00	72.93 ± 0.00	0.56 ± 0.00	8.55 ± 0.00
SM15	1.09	1.96 ± 0.50	27.32 ± 11.71	6.95 ± 1.97	0.17 ± 0.00
SM16	0.32	0.03 ± 0.01	83.32 ± 38.45	2.52 ± 0.64	0.81 ± 0.39
SM17	0.27	0.00 ± 0.00	79.54 ± 0.00	0.54 ± 0.00	0.28 ± 0.00
SM18	0.48	0.02 ± 0.00	80.70 ± 34.76	9.86 ± 0.00	0.18 ± 4.17
SM19	0.46	0.04 ± 0.01	79.49 ± 34.26	10.98 ± 4.30	0.20 ± 0.10
SM20	0.41	0.77 ± 0.24	83.94 ± 32.72	4.64 ± 0.00	0.74 ± 0.32
SM21	1.33	0.37 ± 0.05	56.64 ± 25.50	9.03 ± 2.34	6.43 ± 3.79
SM22	1.52	0.00 ± 0.00	48.98 ± 21.50	6.13 ± 3.06	1.35 ± 0.00
SM23	1.34	0.35 ± 0.14	67.48 ± 23.09	7.33 ± 2.64	2.01 ± 0.75
SM24	1.19	0.41 ± 0.10	69.18 ± 25.12	6.65 ± 2.45	1.43 ± 0.50
SM25	0.57	0.36 ± 0.06	84.55 ± 29.67	1.99 ± 0.93	1.16 ± 0.50
SM26	0.6	0.03 ± 0.00	80.05 ± 34.07	6.97 ± 2.59	5.32 ± 0.40
SM27	0.28	1.34 ± 0.67	77.79 ± 36.21	1.51 ± 0.44	0.52 ± 0.00
SM28	1.23	3.21 ± 0.00	60.88 ± 26.80	11.47 ± 3.04	3.20 ± 0.00
SM29	0.61	0.52 ± 0.26	82.19 ± 28.10	3.40 ± 0.26	0.88 ± 0.00
SM30	1.05	6.31 ± 1.00	57.81 ± 27.85	1.08 ± 0.28	4.52 ± 2.19
SM31	0.21	0.08 ± 0.01	91.04 ± 35.99	0.31 ± 0.09	0.38 ± 0.00
SM32	2.17	10.37 ± 2.34	13.26 ± 3.32	15.47 ± 5.73	0.51 ± 0.00
SM33	2.59	26.86 ± 4.50	1.43 ± 0.53	14.58 ± 1.53	0.00 ± 0.00
SM34	0.926	0.04 ± 0.00	72.93 ± 0.00	0.56 ± 0.00	8.55 ± 0.00
SM35	0.45	0.08 ± 0.02	76.72 ± 30.66	6.82 ± 3.16	0.37 ± 0.16
SM36	0.69	2.33 ± 1.08	74.76 ± 34.22	5.22 ± 0.19	1.80 ± 0.68
SM37	0.3	0.00 ± 0.00	87.64 ± 40.36	2.26 ± 0.27	0.89 ± 0.35
Healthy		34.95 ± 4.62	49.33 ± 14.58	18.35 ± 3.79	4.04 ± 1.31

## Data Availability

The data presented in this study are not publicly available due to ethical restrictions and confidentiality policies.
